# Progress in Surgery

**Published:** 1897-10-02

**Authors:** 


### PROGRESS IN SURGERY.

### ABDOMINAL SURGERY.

### APPENDICITIS.

(*Continued from page 445, Vol. XXII.*)

**Pathology.—Acute Appendicitis.**—It seems clear that the widest difference exists in the rate at which inflammation extends from a focus of infection in the appendix or cæcum, and also in the area which may be involved in each. That the infective lesions themselves are due to bacteria and their products there can be no doubt, for it is possible to demonstrate in such cases their presence in the walls of the appendix, where they are not seen in ordinary conditions, and to find in the fluids of serous and purulent appendicular peritonitis the bacillus coli communis.

Perforation of the appendix is not an essential to peritonitis. The peritoneal lesions come on early, and may be very septic, causing rapid death. In rapidly fatal cases one finds at the autopsy not a peritonitis with abundant purulent fluid, but a sort of subacute peritoneal septicæmia, with some adhesions and a small quantity of pinkish fluid of excessive virulence. In such cases there seems to be a virulent infective parenchymatous interstitial appendicitis. It is said also that an endarteritis obliterans of the branches of the appendicular artery, which pass from the margin of the meso-appendix across the latter structure to supply the appendix, is invariably found. Messieurs Achard and Broca examined the peritoneal fluid in twenty cases of appendicitis in which suppuration had occurred. In seventeen cases the bacillus coli communis was found, and in three it was absent. In one case pneumococci, and in many streptococci were found.

In recurrent and chronic cases there is much fibroid thickening of the muscular coat, with localised peritonitis in the immediate vicinity.

The calculi consist of fæcal matter in 70 per cent. of cases—stercoraceous calculi. They may contain tooth-brush bristles, biliary calculi, ascarides, and all sorts of foreign bodies. The saline constituents are usually calcium phosphate and carbonate, sometimes magnesium sulphate or chloride; and cholesterin may form a basis in rare cases.[6 7 8 9 11 14 16 18 23 34 36]

**Experimental Appendicitis.**—Wherever there is obstruction to a coil of bowel the micro-organisms in the obstructed coil, particularly the bacillus coli, increase in number and virulence, traverse the gut wall, and invade the surrounding peritoneum. This applies to appendicitis.

Roger and Joyué have produced appendicitis artificially in rabbits by ligaturing the appendix of the rabbit without ligaturing the arterial supply. It could be produced by complete ligature, and the absolute transformation of the cavity of the appendix into a closed cyst; no other experimental method would suffice. The authors note that this is not in accordance with the pathology of appendicitis in man determined clinically.[15 36]

**Classification.**—The following two plans appear to be the most generally useful:—

I.

A. With considerable peritonitis.
- (1) Universal peritonitis. No plastic exudate. Probably always fatal.
- (2) Partial peritonitis, limited by plastic exudate or by coaptation of bowel. Sometimes recover.

B. With localised abscess.
- (1) Peritoneal.
- (2) Retrocæcal.

C. Without extra-appendicular abscess.

II.

A. Catarrhal.
- (1) Subacute. } Operation not necessary at first,
- (2) Acute. } but may be advisable later.

B. Suppurative. } Operation.

- (1) Subacute. Forming localised abscess.

- (2) Acute.

  - (*a*) Perforative.

  - (*b*) Gangrenous.

Seeing that gangrenous appendix cannot be accurately diagnosed clinically, the latter classification suggests that there are two indications for operation, viz., (*a*) perforation, (*b*) pus.

**Diagnosis.**—Attention has been called in recent papers to the necessity for differential diagnosis from the following amongst other affections: Abdominal, iliac, psoas, and pelvic abscesses, tubercular peritonitis, hip disease, and gastric ulcer. The pulse, though often related to the temperature, is certainly a more reliable guide. A comparatively low or even subnormal temperature with a pulse of 140 is strongly suggestive of perforative peritonitis. The sudden fall of the temperature with a quickening pulse and increasing symptoms is an intimation that there is no time to lose.[1 2 9]

**Frequency.**—Morris (N.Y.) has estimated that upon an average of two cases of appendicitis per doctor in the United States there must be over 200,000 cases annually in those States.[5 7]

**Mortality.**—There is a strange anomaly here. It is only recently that surgery has dealt with appendicitis so successfully, not only as a curative but as a preventative measure. Dr. North has studied the statistics at Brooklyn, and has found that in spite of the advances of surgery, the deaths from appendicitis and conditions for which it may have been confounded in death certificates, have distinctly increased in percentage of the population, thus: 1880, 1; 1888, $1\frac{1}{4}$; 1893, $1\frac{1}{3}$; 1894, $1\frac{1}{2}$; 1895, $1\frac{1}{3}$. Is appendicitis on the increase, or is the present day treatment less successful? It will be interesting to refer to the table which follows.[5 7 9 11 12 13 16 19 20 21 22 26]

**Prognosis.**—Of the grave signs the following are mentioned: Severe rigors in the onset of the illness during the early days, an increasing rapidity and weakening of the pulse, with a drop in the temperature and increasing abnormal symptoms.[9 27]

**Complications and Sequelæ.**—Among these may be mentioned hepatic abscess, which may occur from appendicitis, even without peritonitis, empyema, hernia of an inflamed appendix, so-called general peritonitis, &c. Some few cases have been recorded of appendicitis complicating pregnancy. Various abscesses may take place from extension, or the infective forms may continue after operation.[2 6 11 28 38 39 40]

**Prediagnostic Treatment.**—In *Treatment* for March,

1897, the late Mr. Greig Smith gave advice as to the prediagnostic treatment of grave abdominal disease. Firstly, give a nutrient enema with an ounce of brandy or whisky, wrap in hot blankets, and cover abdomen with a hot flannel cloth, put hot water bottles to the feet. Secondly, sit down by the bedside to watch the patient and complete the diagnosis, scarcely leaving the bedside till the diagnosis is complete.[1 17]

**Treatment.**—In the routine treatment of appendicitis the intestinal antiseptics—salol and napthaline—are said to be of undoubted value as aids. As regards operation, the average opinion seems to be—(1) Do not necessarily operate for one attack, but watch the patient; (2) in case of a second attack, operate even if the attack subsides favourably, and do not hesitate to remove the appendix in the quiescent period.

The general opinion is that the best incision is a short one, near the right anterior superior spine, and, as far as possible the abdominal muscular fibres should be separated rather than cut, particularly the external oblique. The advantages are that there is less danger of injuring the subjacent intestine, and less tendency to prolapse of omentum and bowel. It enables the surgeon to wall off the peritoneal cavity with ease, and affords good drainage. It avoids infecting the peritoneal cavity, facilitates the removal of the appendix, does less injury to the parietes, and is less likely to give rise to subsequent ventral hernia. It is very necessary to wall off the peritoneal cavity before groping about for the abscess. Trendelenburg's posture may be useful in assisting this procedure. When the abscess has been opened it is generally preferred to sponge out the cavity to avoid the dangers of dissemination by irrigation. If the appendix can be easily found and separated it should be removed, otherwise it is better not to disturb the integrity of the abscess wall. The removal of the appendix takes away the liability to recurrence, and to the formation of a fæcal fistula.

The next question that arises is how best to deal with the stump. The method of inversion of the appendix, as a whole, into the cæcum appears to be dangerous, owing to sloughing of the inverted appendix. Of the other methods there is very little to choose. After ligaturing the meso-appendix (*a*) the stump of appendix may be simply ligatured, or (*b*) a peritoneal cuff may be turned up and afterwards replaced over the ligatured muscle and mucous membrane, or (*c*) a simple ligature may be placed on the stump and then the stump be inverted into the cæcum (after separation of the appendix) by a purse-string or Lambert's suture; or (*d*) the stump may be ligatured close to the cæcum and a part of the cæcum be inverted. The stump is usually treated with a strong antiseptic to avoid infection.

It is unnecessary to drain in the catarrhal forms (recurrent), but in suppurative cases, india-rubber or glass tubes or gauze drainage are requisite.

Whether to operate is an anxious question. Dr. Mereness gives the following indications for operation: (1) When there is pus, (2) perforation, (3) generalised peritonitis, (4) reasonable time allowed without recovery, (5) relapses. Against operation he places—(1) Simple acute or chronic catarrhal appendicitis, (2) localised peritonitis, (3) diffuse septic peritonitis.

Mr. Mayo Robson rather favours the American doctrine of operating as soon as appendicitis is diagnosed, and thinks there would thus be a greater percentage of recoveries, considering the safety of the operation in so-called catarrhal appendicitis.

As regards the time for operation, in the acute suppurative forms, some surgeons say, "The sooner the better"; others wait for the limiting adhesions to become sound. In recurrent appendicitis all are agreed that the operation should take place in the quiescent interval between attacks. Many American surgeons extend operation also to the first acute non-suppurative cases ("catarrhal").

Shrady's (New York) rules for operation represent fairly an average opinion: (1) Progressively accelerating pulse; (2) progressive pain; (3) increase of temperature, associated with the foregoing. He prefers to wait for adhesions to become firm in case of abscess. McBurney, however, urges operation as soon as an abscess is diagnosed.[1 2 5 6 9 11 13 16 18 19 22 23 24 25 27 28 29 30 32 34]

**Cases of Diffuse Peritonitis, Generalised or General.**—It is necesasry to distinguish between universal and generalised peritonitis. A few years ago one or two cases of so-called general septic peritonitis were recorded as having recovered after operation; and it has now become the fashion for surgeons to call general that which is truly only partial—it looks better on paper. Recovery from a universal septic peritonitis must still be a very rare event, indeed.

Under this heading successful cases have been recorded recently by Mr. Turner, of Sussex; Messrs. Toller and Wallace, of St. Thomas's Hospital; and Messrs. Mayo Robson and Moynihan, of Leeds; as well as by Dr. Abbe, of America.[30 32 33 35 39]

**Bibliography, &c.**—For recent literature on the subject of appendicitis the reader may also refer to the summary in the *Medical Chronicle* for April, 1897.

[1] Malcolm, Practitioner, May. 1897. [2] Wright, Amer. Med.-Surg. Bulletin, Mar. 10, 1897. [3] Page, Clinical Journ., April 21, 1897. [4] Payne, Charlotte Med. Journ. [5] Medical Week, Dec. 11, 1896. [6] Barker, Practitioner, Feb. 1897. [7] Morris, N. Y. Med. Record, Dec. 26, 1896. [8] Robinson, Annals of Surg., Dec, 1896. [9] MacDougall, Edin. Med. Journ., Dec., 1896. [10] Edin. Med. Journ., Dec., 1896. [11] Dieulafoy, Clin. Journ., Sept. 16, 1896. [12] N. Y. Med. Record, Oct, 24, 1896. [13] Glasgow Med. Journ., Sept., 1896. [14] Med. Week, Dec. 18, 1896. [15] Quarterly Med. Journ., Oct., 1896. [16] Hare, Australasian Med. Gaz., July 20, 1896. [17] N. Y. Med. Record, April 24, 1897. [18] Lancet, April 3, 1897. [19] N. Y. Med. Record, Dec. 12, 1896. [20] N. Y. Med. Record, Oct. 17, 1896. [21] N. Y. Med. Record, Sept. 26, 1896. [22] N. Y. Med. Record, Oct. 10, 1896. [23] Amer. Journ. Med. Science, Feb., 1897. [24] N. Y. Med. Record, Jan. 9, 1897. [25] Amer. Med.-Surg. Bulletin, Mar. 10, 1897. [26] N. Y. Med. Record, Dec. 19, 1896. [27] N. Y. Med. Record, Jan. 9, 1897. [28] N. Y. Med. Record, Oct. 24, 1896. [29] Birmingham Med. Review, Dec., 1896. [30] Lancet, Dec. 19, 1896. [31] Lancet, Dec. 19, 1896. [32] Lancet, Dec. 26, 1896. [33] Brit. Med. Journ., Dec. 12, 1896. [34] Brit. Med. Journ., Dec. 19, 1896. [35] Lancet, Nov. 7, 1896. [36] Medical Week, Oct. 16, 1896. [37] N. Y. Med. Record, Oct. 17, 1896. [38] Charlotte Medical Journ. [39] N. Y. Med. Record, Mar. 6, 1897. [40] N. Y. Med, Record, Sept. 26, 1896. [41] Med. Chronicle, April, 1897.

